# Vacuolar Membrane ATPase Activity 21 Predicts a Favorable Outcome and Acts as a Suppressor in Colorectal Cancer

**DOI:** 10.3389/fonc.2020.605801

**Published:** 2021-02-19

**Authors:** Fan Zhang, Hao Shen, Yating Fu, Guanyu Yu, Fuao Cao, Wenjun Chang, Zhongdong Xie

**Affiliations:** ^1^ Department of Environmental and Occupational Health, Second Military Medical University, Shanghai, China; ^2^ School of Medicine, Yunnan University, Kunming, China; ^3^ Department of Gastrointestinal Surgery, First Affiliated Hospital of Wenzhou Medical University, Zhejiang, China; ^4^ Department of Colorectal Surgery, Changhai Hospital, Second Military Medical University, Shanghai, China; ^5^ Department of Colorectal Surgery, Union Hospital, Fujian Medical University, Fuzhou, China

**Keywords:** vacuolar membrane ATPase activity 21 (VMA21), vacuolar ATPases, prognosis, tumor suppressor, colorectal carcinoma

## Abstract

Extracellular and/or intracellular manipulation of pH in tumor may have noticeable potential in cancer treatment. Although the assembly factor genes of V_0_ domain of the V-ATPase complex are required for intracellular pH homeostasis, their significance in colorectal cancer (CRC) remains largely unknown. Here, we used bioinformatics to identify the candidates from known assembly factor genes of the V_0_ domain, which were further evaluated by immunohistochemistry (IHC) in CRC and adjacent normal specimens from 661 patients. Univariate and multivariate Cox analyses were used to evaluate factors contributing to prognosis. The effects of variations in the expression of VMA21 on tumor growth were assessed *in vitro* and *in vivo*. Of five known assembly factors, only VMA21 showed differential expression between CRC and adjacent normal tissues at both mRNA and protein levels. Patients with high VMA21 expression had higher differentiation grade and longer disease-specific survival (DSS) at stages I–III disease. High VMA21 expression in tumors was also an independent predictor of DSS (hazard ratio, 0.345; 95% confidence interval, 0.123–0.976), with covariates included TNM stage and differentiation grade. VMA21 overexpression decreased CRC growth, whereas VMA21 knockdown increased CRC growth *in vitro* and *in vivo*. VMA21 expression suppresses CRC growth and predicts a favorable DSS in patients with stage I-III disease.

## Introduction

Colorectal cancer (CRC) is one of the most common malignances worldwide, and surgical resection supplemented with chemotherapy remains the most effective treatment in patients without distant metastasis (1, 2). However, 30–50% of patients experience relapse or metachronous metastases after surgery (1, 2). Post-surgical chemotherapy and extensive surveillance are beneficial for patients who are more likely to show disease exacerbation. However, identifying patients who would benefit from these treatments is challenging because tumors are heterogenous, even among patients with the same tumor-node-metastasis (TNM) stage (1–3). The only validated marker for prognosis stratification and for selecting the appropriate chemotherapy in CRC is microsatellite instability (MSI) (3, 4), although many biomarkers have been investigated. Therefore, additional effective biomarkers are urgently required (5).

The vacuolar H+-ATPase complexes (V-ATPases) are large multisubunit protein complexes that are required to maintain the pH homeostasis of intracellular compartments (6). The complex consists of two domains, a membrane-integral V_0_ domain for proton translocation and a cytosolic V1 domain for ATP hydrolysis, which are assembled separately in the endoplasmic reticulum and cytosol, respectively (7, 8). The assembly of the V_0_ domain depends on a set of endoplasmic reticulum (ER)-resident chaperones, including TMEM199, VMA21, CCDC115, ATP6AP1, and ATP6AP2 (8). Loss-of-function mutations of the assembly factor genes are associated with a spectrum of disease symptoms (8–13), and the normal function of CCDC115, ATP6AP1, and ATP6AP2 is associated with favorable phenotypes in several cancer types (14, 15). However, the clinical significance and biological role of assembly factor genes in CRC is largely unknown.

To address these challenges, in the study we aimed to evaluate whether the five assembly factor genes of V_0_ domain associated with the development and progression of CRC and further to investigate the biological function of the candidate. We showed that among five V_0_ domain assembly factors genes, VMA21 was the only differentially expressed gene between CRC and neighboring normal tissues. Tumors with high VMA21 expression had higher differentiation grade and were associated with longer disease-specific survival (DSS) than VMA21-low tumors. Ectopic expression of VMA21 reduced the growth of CRC cells *in vitro* and *in vivo*. The present data suggest that VMA21 acts as a tumor suppressor and predicts a favorable prognosis, although VMA21 expression is elevated in CRC tissues.

## Materials and Methods

### Bioinformatics Analysis

The expression of five genes (TMEM199, VMA21, CCDC115, ATP6AP1, and ATP6AP2) in CRC and corresponding normal tissue specimens was firstly analyzed on the Expression Profiling Interactive Analysis (GEPIA) website based on the dataset of The Cancer Genome Atlas (TCGA) (16). Co-expression of the genes was analyzed using the Pearson Correlation Coefficient (PCC). Candidate genes showing a 1.5-fold difference between CRC and adjacent normal tissues were further investigated in other cancer types using TCGA data and in our CRC cohort by immunochemistry (IHC).

### Patients and Tissues

Formalin-fixed, paraffin-embedded (FFPE) specimens from 661 cancerous tissues, 26 adenoma, and 65 para-carcinoma tissues were obtained from 661 patients treated at Changhai Hospital, Second Military Medical University, Shanghai, between 2001 and 2011. None of the patients received chemotherapy or radiation therapy before surgery. All tissue samples were tested histologically by the pathologists to verify the diagnosis. Outdo Biotech Co., Ltd (Shanghai, China) constructed the tissue microarrays (TMAs) containing the FFPE specimens as previously described (17). The baseline information of each specimen donors, including age, tumor location, differentiation grade, number of examined lymph nodes, TNM stage (determined according to the American Joint Committee on Cancer Staging Manual, seventh edition), adjuvant chemotherapy, serum carcinoembryonic antigen (CEA), and carbohydrate antigen 199 (CA199) levels, were documented. The follow-up of patients was performed regularly as previously described (17). The disease-specific survival (DSS) and disease-free survival (DFS) were defined as the time from surgery to death caused by CRC and the time from surgery to relapse, respectively. Written informed consent was obtained from all patients and approved by the Ethics Committees of Changhai Hospital.

### Immunohistochemistry

The slides of TMAs (4-μm thick) were prepared for IHC examination of the detection of VMA21 immunstaining. Each slide was deparaffinized using xylene and rehydrated in graded alcohol, and endogenous peroxidase activity was blocked using 3% H_2_O_2_. After antigen retrieval of VMA21 for 30 minutes in 10 mmol/L sodium citrate buffer (pH 6.0), slides were blocked with 5% normal goat serum for 10 min at room temperature. Then, the slides were incubated with anti-human VMA21 polyclonal antibody (PA5-42630, 1:150 dilution; Invitrogen, Carlsbad, California, USA) overnight at 4°C, followed by incubation with a secondary antibody from the ElivisionTMsuper HRP (Mouse/Rabbit) for 30 min. Then, the sections were reacted with 3-3-aminobenzidine (DAB) solution for 45 seconds and counterstained with hematoxylin for 25 seconds.

### Immunohistochemistry Scoring

Two authors (F.Z. and H.S.) who were blinded to the clinicopathological information, evaluated the VMA21 immunostaining data independently. The intensity level of each specimen dot was evaluated using the H-score method as described previously (18, 19). H-score was evaluated by multiplying the average percentage of positive cells (0–100%) by the staining intensity (0, negative; 1, weakly positive; 2, moderately positive; and 3, strongly positive staining). The IHC scores of each specimen provided by the two observers were averaged for further studies.

### Cell Culture, qRT-PCR, and Western Blotting

Four human CRC cell lines (RKO, SW620, LoVo, and CACO2) were maintained in RPMI-1640 or DEME medium with 10% heat-inactivated fetal bovine serum (FBS) (GIBCO, Grand Island, NY, USA) and 2% penicillin/streptomycin (GIBCO). All cells were obtained commercially from the Cell Bank affiliated to the Chinese Academy of Sciences (Shanghai, China). The LightCycler 480 II system (Roche, Basel, Switzerland) with SYBR Green reagent (Takara, Bio, Dalian, China) was used to detect the relative expression of *VMA21* mRNA using the following primers: 5′-TGG AGC GCC CGG ATA AGG-3′ (forward) and 5′-CTG TTT GCC TTC ACG CCA C-3′ (reverse). Human GAPDH served as an internal control with the primers 5′-GGA GCG AGA TCC CTC CAA AAT-3′ (forward) and 5′-GGC TGT TGT CAT ACT TCT CAT GG-3’ (reverse). Western blot analysis of CRC cells and specimens was performed using routine protocols with antibodies against human VMA21 (1:150, PA5-42630, Invitrogen) or human GAPDH (1:1000, AP0063, Bioworld Technology, St. Louis Park, MN, USA)

### cDNA Expression Constructs

Invitrogen Co., Ltd (Shanghai, China) synthesized and validated the cDNA sequence of the VMA21 transcript (NM_001017980). The products were subcloned into the pENTRTM3C vector, and the recombinant VMA21 was Gateway-recombined into the pInducer 20 vector (Addgene) (20). A Lenti-XTM HTX Packaging System (Clontech Laboratories, Inc., CA, USA) was used to produce lentiviral particles in HEK293T cells, which were titrated onto SW620 or LoVo cells cultured in media containing 1 µg/mL puromycin to reach the optimal expression of the target protein using the minimum viral load. G418 (2 mg/ml) was added to induce *VMA21* gene expression, which was confirmed by qRT-PCR and western blotting.

### Construction of Stable Cells With Inducible *V*acuolar *M*embrane ATPase *A*ctivity 21 Knockdown

A microRNA (miR)-30 loop and appropriate flanking sequences were added to the different miR-30-mediated shRNAs to specifically target VMA21 as described previously (21). The sequence was as followed: 5′-CAU CUA CAC UGA AGA CGC UTT AGC GUC UUC AGU GUA GAU GTT-3′. After the synthesis of single-stranded DNA templates for shRNAs, the templates were amplified with primers including Xho1 or BamH1 restriction enzymes sites. The target DNA was purified and subcloned into the Pinducer10 vector (20). Lentiviral particles were produced as described above and titrated onto RKO cells. Expression of VMA21 was induced using 1 μg/mL doxycycline (dox) and confirmed by qRT-PCR and western blotting.

### Colony Formation Assay

CRC cells (LoVo and SW480) were seeded into 6-well plates (Corning, NY, USA) at a density of 1.5 × 10^3^ cells/well. The cell medium was replaced every 72 h during growth. After 7–14 days of incubation, the supernatant was removed, cells were fixed with methanol, and then dyed with crystal violet. Images were captured, and colonies were counted when they contained more than 50 cells.

### *In Vivo* Tumor Growth

Male nude mice (BALB/c, 5–6 weeks old) were purchased from SLAC Laboratory Animal Co. (Shanghai, China) and were accustomed to a pathogen-free environment for 1 week. For setting up the xenograft implant tumor models, the stable inducible CRC cells (VMA21-LoVo, VMA21-SW620, and shRNA-VMA21 RKO) were subcutaneously injected into test (treated with G418 or Dox) and control groups. Tumor formation was continuously monitored using calipers every 3–4 days. The tumor volume was calculated using the formula (length × width^2^)/2. The mice were euthanized after 19–30 days depending on tumor size, and the tumor xenografts were removed and tested. All procedures were performed according to the National Research Council’s Guide for the Care and Use of Laboratory Animals.

### Statistical Analysis

Differences in VMA21 expression between CRC and adjacent non-tumorous tissues were analyzed using the Student’s *t* test and we used independent-sample t-tests for the differences of mRNA expression in different CRC cells. The χ2 test and Student’s *t* test were used to compare categorical data and continuous data separately. The R package of maxstat (22) was used to determine the optimal value for the classification of patients into subgroups according to the expression of VMA21 determined by immunostaining. The subgroups were further compared using the Kaplan-Meier method and Cox proportional hazards models. All statistical tests were performed with R 3.6.1 and SPSS (Version 23.0 for Windows), and two-tailed tests with a *P <*0.05 were considered significant.

## Results

### Vacuolar Membrane ATPase Activity 21 is Upregulated in Colorectal Cancer Epithelial Cells

**Figure 1:**
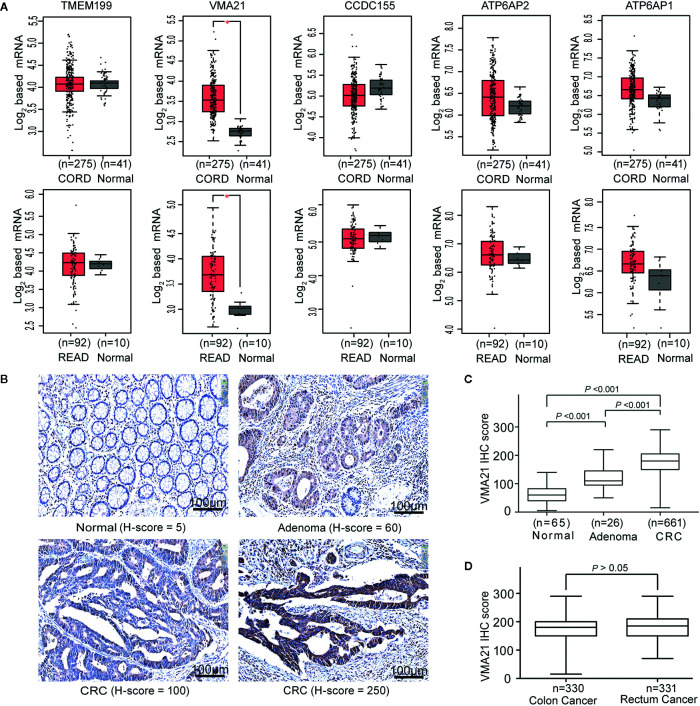
VMA21 is elevated in colorectal cancer. **(A)** Expression patterns of five assembly factor genes of the V_0_ domain of V-ATPase based on TCGA-CRC mRNA data. **(B)** Immunostaining of VMA21 in CRC and noncancerous tissues. **(C)** VMA21 protein expression in normal, adenoma, and CRC tissue specimens. **(D)** Expression pattern of the VMA21 protein in colon cancer and rectal cancer. CORD, colon cancer; READ, rectum cancer.

To identify V-ATPase assembly factor gene candidates associated with the progression of CRC, the expression of five genes required for the V_0_ domain of V-ATPase (TMEM199, VMA21, CCDC115, ATP6AP1, and ATP6AP2) (8) were analyzed using TCGA-CRC cohort. Three (CCDC115, ATP6AP1 and ATP6AP2) of the five assembly factor genes are associated with several cancers (14, 15). We found that VMA21 was the only gene showing significantly higher mRNA expression in colon and rectal cancerous tissues than in adjacent normal tissues (all *P* < 0.05), as shown in **Figure 1A**. The associations between VMA21 and ATP6AP1 (r = 0.56, *P* < 0.001) and ATP6AP2 (r = 0.52, *P* < 0.001) were significant, as indicated by Pearson correlation coefficients (**Supplementary Figure 1**). Therefore, VMA21 was identified as a new candidate gene involved in the development of CRC.

VMA21 expression in CRC epithelial cells may be affected by the expression profiles of whole tissues because of the potential mixture of transcripts from different cell populations (23, 24). We therefore investigated the expression pattern of VMA21 in our specimens using IHC. VMA21 protein expression was primarily detected in the cytoplasm of colorectal epithelial cells (**Figure 1B**). The immunostaining marks of VMA21 increased gradually in adjacent normal tissues, adenoma, and primary CRC (*P trend* < 0.001), as shown in **Figure 1C**. There was no difference in VMA21 protein expression between colon and rectal cancerous tissues (**Figure 1D**, *P >* 0.05). Taken together, these results indicate that VMA21 is upregulated in CRC at the mRNA and protein levels, suggesting that it plays a role in CRC.

### Associations Between *Vacuolar Membrane ATPase Activity 21* Expression and Clinical Variables of C*olorectal Cancer* Patients

Based on the 97.5% quantile (175) of VMA21 in the IHC scores from noncancerous specimens, the 661 patients were classified into two groups, the VMA21-high group (score >175) and the VMA21-low group (score ≤ 175). The associations between VMA21 expression and the clinical features of CRC patients are shown in **Table 1**. There were no significant associations between VMA21 protein expression and several clinical factors (all *P >*0.05), including age, gender, TNM stage, tumor location, and serum CEA and CA199 levels. However, VMA21-high expression was significantly associated with higher differentiation grade (*P* = 0.011) compared with VMA21-low expression, indicating that the expression of VMA21 may be negatively related to the progression of CRC.

**Table 1:** Characteristics of patient with CRC dichotomized by VMA21 expression.

Characteristics	Expression of VMA21 protein		*P* Value*
	Low(n = 289)	High(n = 372)	
**Age (years)**	61.47 ± 12.77	60.19 ± 13.02	0.208**
**Differentiation grade, n (%)**			**0.011**
Well+ Moderately	214 (74.0)	308 (82.8)	
Poor	65 (22.5)	56 (15.1)	
Missing	10 (3.5)	8 (2.1)	
**Lymph nodes, n (%)**			0.677
≤ 12	89 (30.8)	109 (29.3)	
>12	200 (69.2)	263 (70.7)	
**TNM stage, n (%)**			0.211
I+II	187 (64.7)	223 (59.9)	
III+ IV	102 (35.3)	149 (40.1)	
**Chemotherapy, n (%)**			0.369
Yes	226 (78.2)	281 (75.6)	
No	49 (17.0)	50 (13.4)	
Missing	14 (4.8)	41 (11.0)	
**Serum CEA, n (%)**			0.684
< 5ng/ml	177 (61.2)	224 (60.2)	
≥ 5ng/ml	108 (37.4)	145 (39.0)	
Missing	4 (1.4)	3 (0.8)	
**Serum CA199, n (%)**			0.960
< 37U/ml	242 (83.7)	313 (84.1)	
≥ 37U/ml	43 (14.9)	55 (14.8)	
Missing	4 (1.4)	4 (1.1)	

*Pearson’s chi-squared test.

**Student’s t test.

Missing values are excluded for all statistic tests.

CRC, colorectal cancer; CEA, carcinoembryonic antigen; CA199, carbohydrate antigen 199.

Bold number represents statistically p value < 0.05.

### High Vacuolar Membrane ATPase Activity 21 Expression Tends to Indicate a Favorable Outcome

**Figure 2:**
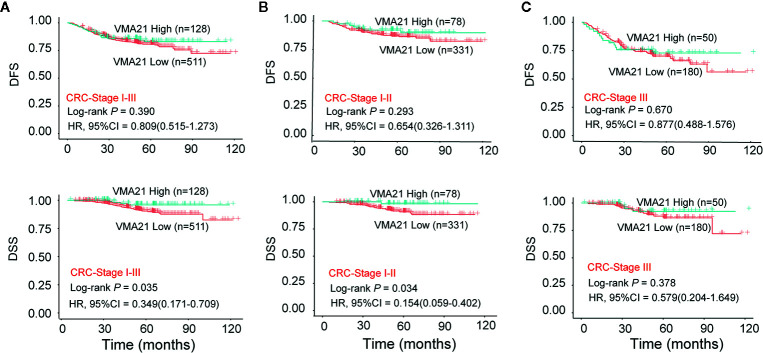
Relationship between VMA21 expression and patient survival. **(A–C)** DFS and DSS analysis in patient subgroups with high or low VMA21 protein expression in stage I-III, stage I-II, and stage III disease. DFS, disease free survival; DSS, disease specific survival.

The associations between the expression of VMA21 and clinical outcomes were further investigated in 639 patients with stage I-III CRC. According to the optimal IHC-score cut-off value (215) determined by maxstat software, which could most efficiently distinguish differences in clinical outcomes (**Supplementary Figure 2**), patients were classified into two subgroups: patients with VMA21-high (>215) and those with VMA21-low (≤215) tumors. As shown in **Figure 2A**, VMA21-high tumors were strongly correlated with a favorable DSS (*P* = 0.035) but not DFS (*P* = 0.390). Next, the associations between VMA21 expression and DFS and DSS in patients with stage I and II disease were estimated. The results showed a significant association between VMA21 expression and DSS (*P* = 0.034), but not DFS, in stage I-II CRC patients (**Figure 2B**). VMA21 expression was not significantly associated with DFS or DSS in patients with stage III disease (*P* > 0.05) (**Figure 2C**). Multivariate Cox analysis (**Table 2**) showed that VMA21 expression was associated with DSS [hazard ratio (HR), 0.345; 95% confidence interval (CI), 0.123–0.976] in patients with stage I–III disease, with the covariates including stage, lymph nodes, grade, and serum CEA levels. In patients receiving chemotherapy, the expression of VMA21 in CRC was marginally (*P* = 0.062) associated with DSS in stage II disease, whereas the association was not significant in patients who did not receive chemotherapy (*P* = 0.52) (**Supplementary Figure 3**).

**Table 2:** Cox regression analysis of prognostic factors for DSS in 639 CRC patients.

Variables	Univariate analysis		Multivariate analysis*	
	HR (95% CI)	*P* value	HR (95% CI)	*P* value
**VMA21 scores**				
High vs. Low	0.340 (0.122–0.950)	**0.039**	0.345 (0.123–0.976)	**0.043**
**Gender**				
Male vs. Female	1.202 (0.662–2.184)	0.545		
**Tumor location**				
Colon vs. Rectum	1.058 (0.589–1.903)	0.850		
**Differential grade**				
Per increase in grade	1.904 (1.007–3.597)	**0.047**	1.649 (0.847–3.210)	0.142
**Lymph nodes**				
>12 vs. ≤12	1.916 (0.969–3.785)	0.061	2.075 (1.046–4.117)	**0.037**
**TNM stage**				
Per increase in stage	1.511 (0.939–2.431)	0.089	1.385 (0.848–2.262)	0.193
**Chemotherapy**				
Yes vs. No	1.766 (0.627–4.977)	0.282		
**Serum CEA (ng/mL)**				
< 5 vs. ≥ 5	0.542 (0.302–0.973)	**0.040**	0.603 (0.333–1.090)	0.094
**Serum CA199 (U/mL)**				
< 37 vs. ≥37	0.692 (0.322–1.486)	0.345		

*Variates with a p value of <0.1 in univariate analysis were included in the multivariate analysis.

CRC, colorectal cancer; DSS, disease specific survival; CA199, carbohydrate antigen 199; CEA, carcinoembryonic antigen; TNM, tumor to node to metastasis.

Bold number represents statistically p value < 0.05.

### Vacuolar Membrane ATPase Activity 21 Inhibits the Growth of Colorectal Cancer Cells

**Figure 3:**
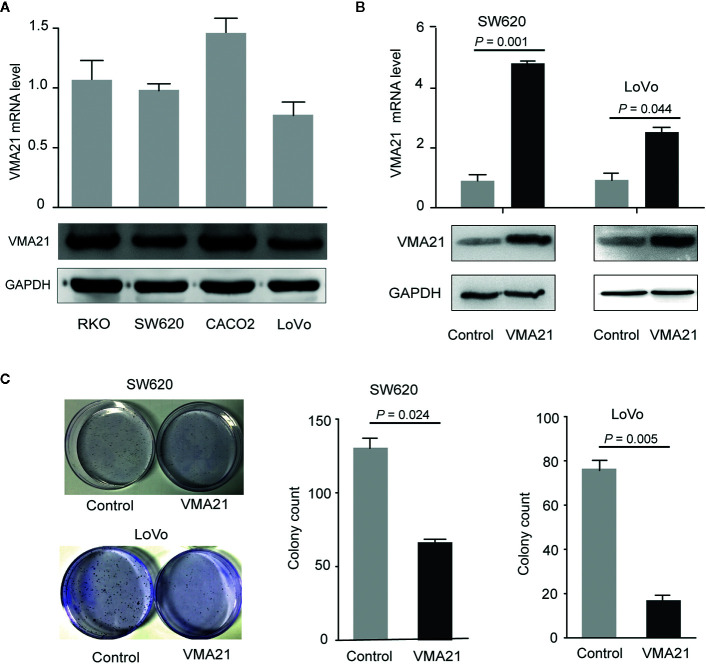
VMA21 overexpression suppresses colony formation of CRC cells. **(A)** Baseline mRNA and protein expression of VMA21 (lower panel) in four CRC cell lines. **(B)** The efficiency of VMA21 overexpression in the indicated CRC cells at the mRNA and protein levels (lower panel). **(C)** The colony formation abilities are affected by overexpressed VMA21 in CRC cells.

The effect of VMA21 expression on the growth of CRC cells was tested to explore. As shown in **Figure 3A**, VMA21 expression is relatively low in LoVo and SW620 cells, and VMA21 was overexpressed in these cell lines for further evaluation (**Figure 3B**). In the colony formation assays, VMA21 overexpression decreased the number of CRC cell colonies compared with those in the control groups in both SW620 and LoVo cell models (**Figure 3C**). The data suggest that VMA21 is involved in the negative regulation of CRC cell proliferation.

### Vacuolar Membrane ATPase Activity 21 Suppresses Colorectal Cancer Growth in Animal Models

**Figure 4:**
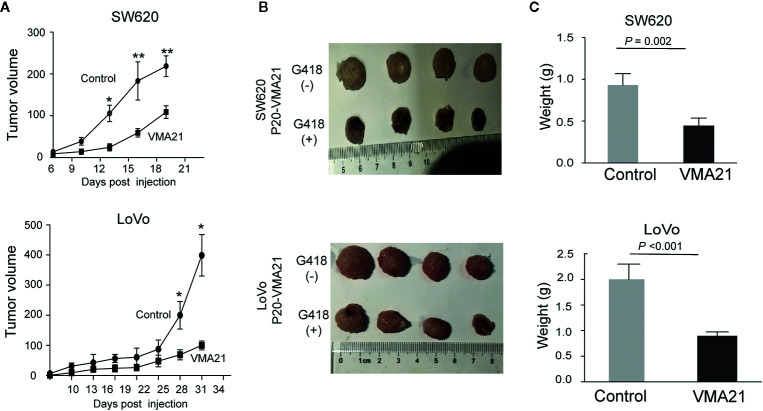
Effects of VMA21 overexpression on xenograft tumor growth. **(A)** Dynamic effect of VMA21 overexpression on the volume of xenograft CRC models. **(B)** Isolated tumors after surgical excision. **(C)** Comparison of the weights of isolated tumors between the subgroups of VMA21 overexpression and the control. (*P < 0.05; **P < 0.01; ***P < 0.001).

**Figure 5:**
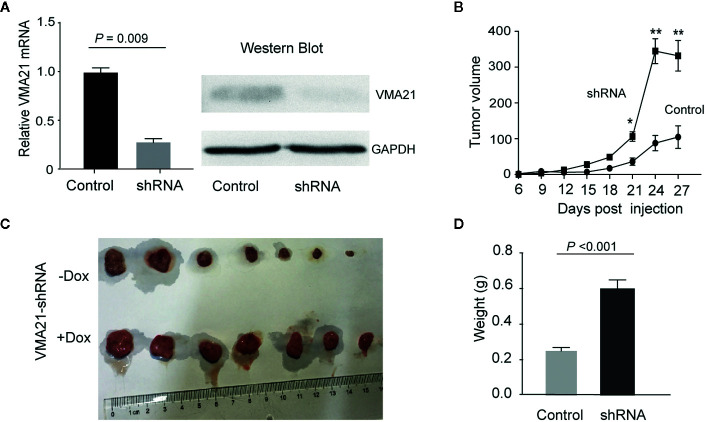
Effects of VMA21 knockdown on xenograft tumor growth. **(A)** The efficiency of VMA21 knockdown in RKO cells at the mRNA and protein levels. **(B)** Dynamic effect of VMA21 knockdown on the volume of CRC xenograft models. **(C)** Isolated tumors after surgical excision. **(C)** Comparison of the isolated tumor weights from the subgroups with VMA21 knockdown or not. (*P < 0.05; **P < 0.01; ***P < 0.001).

VMA21-SW620 or VMA21-LoVo cells were subcutaneously injected into BALB/c nude mice (supplemented with or without G418 in drinking water) to determine whether VMA21 overexpression reduces CRC growth in animal models. In the first 10 days for VMA21-SW620 and the first 25 days for VMA21-LoVo models, tumor size did not differ significantly between the test (G418+) and control (G418-) groups in both cell models (**Figure 4A**). However, xenograft tumors were significantly smaller in the test groups than in the control groups after the indicated days (**Figure 4A**). By the end of the observation period, both the size (**Figure 4B**) and weight (**Figure 4C**) of isolated tumors were significantly lower in the test animals than in the control animals for both cell models. In xenograft tumors from RKO cells, knockdown of VMA21 promoted the development of CRC, as shown in **Figure 5**. Therefore, high VMA21 expression suppresses tumor growth *in vivo*, indicating that VMA21 is a negative regulator of CRC tumorigenicity.

## Discussion

The lysosome mediates the degradation and recycling of macromolecules and signals to the cytosol and nucleus by releasing metabolites and ions; it is implicated in several malignancies (25). These lysosomal behaviors depend on a low intraluminal pH. Although the V_0_ domain of V-ATPases is required for proton translocation and lysosome acidification (6–8), the role of the assembly factor genes of the domain in cancer remains largely unknown. In this study, we identified VMA21 as the only candidate gene showing differential expression between cancer and noncancerous colorectal tissues among five known assembly factor genes (TMEM199, VMA21, CCDC115, ATP6AP1, and ATP6AP2) of the V_0_ domain. IHC data confirmed that VMA21 expression is higher in CRC than in adjacent normal tissues. The data consistently suggested that VMA21 plays a critical role in the development of CRC.

The genes encoding CCDC115, ATP6AP1, and ATP6AP2 may play a tumor suppressor role in different cancer types (14, 15). In this study, the expression of VMA21 was correlated with the expression of ATP6AP1 and ATP6AP2, although which were not differentially expressed between cancer and noncancer tissues. These data suggest that VMA21 plays a negative role in CRC development. Similar findings were reported for the gene encoding GUCY2C, which is elevated in CRC but serves as a tumor suppressor (26). Further, we observed that high expression of VMA21 was associated with the well differentiation of CRC. Survival analysis showed that high expression of VMA21 was associated with longer DSS in stage I–III disease and served as an independent risk factor, and this observation was similar in patients with early CRC and in those receiving chemotherapy for stage II disease. Although we observed no associations between DFS and the expression of VMA21 in the subgroups with different disease stages or chemotherapy regimens, the evidence indicated that elevated VMA21 may be a important factor involved in controlling the progression of CRC.

To further elucidate the biological role of VMA21 in CRC, we modulated the expression of VMA21 in different CRC cells and evaluated its function. Overexpression of VMA21 in human colon cancer LoVo and SW620 cells significantly suppressed colony formation ability. Consistently, ectopic expression or knockdown of VMA21 significantly inhibited or increased CRC development in animal models, confirming the clinical data and *in vitro* results, as well as the suppressive effect of VMA21 on CRC growth. Previous investigation of the underlying molecular mechanism showed that VMA21 deficiency decreases lysosomal-mediated degradation and blocks autophagy (27), and increased autophagy inhibits tumor progression (28, 29). However, the recent evidence also shows that VMA21 may have a positive role to promote the growth of ovarian cancer and lung cancer cells (30, 31). Therefore, tumorigenesis is an extremely complex process involving several factors and many biological phenomena, and it reflects a competition between carcinogens and tumor suppressors (32). Defining the exact role of VMA21 in CRC requires further investigation.

The present study had several limitations. First, the inconsistencies in the prognostic role of VMA21 in DFS and DSS cannot be explained. Second, other critical prognostic factors, such as MSI (4) were not included in our studies because the information was difficult to obtain. Finally, the potential mechanism about the role VMA21 as a tumor suppressor was not explored in the present study. In summary, we showed that VMA21 is upregulated in CRC and associated with a high differentiation grade and favorable DFS. Ectopic expression of VMA21 in CRC significantly inhibited the growth of CRC in cell culture models and animal models. Although the mechanisms involved in VMA21-mediated tumor suppression remain unclear, the current study provides the first evidence of the clinical and biological significance of VMA21 in CRC, as well as their application in the targeted drugs and chemotherapy will be further explored based on mechanism research. The expression of VMA21 may represent a potential diagnostic and prognostic marker for CRC, especially for patients with early-stage CRC.

## Data Availability Statement

The raw data supporting the conclusions of this article will be made available by the authors, without undue reservation.

## Ethics Statement

The studies involving human participants were reviewed and approved by the Ethics Committees of the Changhai Hospital. The patients/participants provided their written informed consent to participate in this study. The animal study was reviewed and approved by the Ethics Committees of the Changhai Hospital.

## Author Contributions

As for the authorship, FZ, HS, and YF analyzed whole data independently and presented the same results. GY and FC were responsible for the follow-up of CRC patients. FZ, HS, and YF were responsible for pathological analysis. GY, FC, and ZX were involved in the pathological diagnosis and recruitment of the patients in the hospital. FZ, WC, and ZX were responsible for the statistical analysis. WC, FZ, and ZX designed and organized the study and wrote the manuscript. All authors contributed to the article and approved the submitted version.

## Funding

This work was supported by grants from the National Natural Science Foundation of China (81972302 and 81572451 to WC) and the Wenzhou Science & technological Project (Y2020927 to ZX).

## Conflict of Interest

The authors declare that the research was conducted in the absence of any commercial or financial relationships that could be construed as a potential conflict of interest.
