# Supplementary material for: Vacuolar Membrane ATPase Activity 21 Predicts a Favorable Outcome and Acts as a Suppressor in Colorectal Cancer
Source: Front Oncol. 2021 Feb 19;10:605801. doi: 10.3389/fonc.2020.605801 (PMC7933500; doi:10.3389/fonc.2020.605801)
Supplement: Supplementary file 1 [file DataSheet_1.doc]

**Data Supplement**

**Title:**

**Vacuolar membrane ATPase activity 21 (VMA21) predicts a favorable outcome and acts as a** **suppressor in colorectal cancer**

Supplementary Figure S1

Supplementary Figure S2

Supplementary Figure S3

**Supplementary Figure S1**

**Supplementary Figure S1. The associations between** **VMA21 and each of ATP6AP1, ATP6AP2, CCDC115 and TMEM199 genes.**

**Supplementary Figure S2**

*
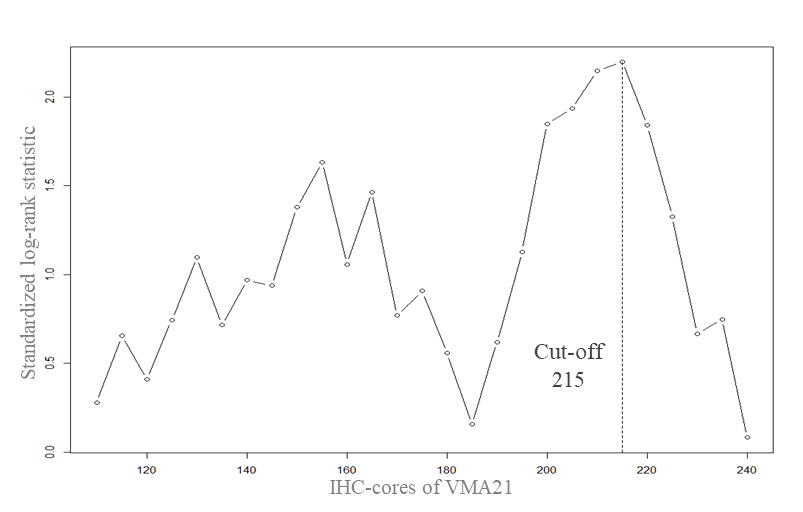
*

**Supplementary Figure S2: The highest absolute standardized log-rank statistics is reached as IHC-score of 215 used to define patient subgroups with high or low VMA21.**

**Supplementary Figure S3**

**Supplementary Figure S3. Associations between VMA21 expression and survival of stage II CRC patients with or without chemotherapy.**
